# Factors perceived to influence implementation of task shifting in highly specialised healthcare: a theory-based qualitative approach

**DOI:** 10.1186/s12913-018-3719-0

**Published:** 2018-11-27

**Authors:** Eli Feiring, Astrid Eidesvik Lie

**Affiliations:** 1 Department of Health Management and Health Economics, University of Oslo, PO Box 1089, Blindern, 0317 Oslo, Norway; 2 Division of Laboratory Medicine, Oslo University Hospital, PO Box 4950, Nydalen, 0424 Oslo, Norway

**Keywords:** Implementation, Specialised healthcare, Task shifting, Qualitative, Bone-marrow examination

## Abstract

**Background:**

New approaches to control healthcare expenditures and increase access to quality care are required by decision-makers in high-income countries. One strategy is to reallocate tasks from doctors to nurses. Evidence suggests that quality, effectiveness and efficiency of task shifting are context sensitive and affected by implementation. However, little is known about implementability of task shifting in specialised healthcare. We aimed to identify factors perceived to influence implementation of doctor-to-nurse task shifting in a hospital setting and improve understanding of task shifting implementability by using theory-based frameworks for analysing behaviour. Nurse-led bone marrow examination exemplified task shifting from the medical to the nursing domain.

**Methods:**

Doctors and nurses (*n* = 17) in a haematology department at a Norwegian university hospital were asked about factors perceived to influence implementation of nurse-led bone marrow aspirations and biopsies. Methods included in-depth semi-structured interviews (*n* = 11) and focus-group discussion (*n* = 6). Data were analysed using the Capability, Opportunity, and Motivation behaviour model and the Theoretical Domains Framework.

**Results:**

Ten factors perceived to influence implementation were identified. Three factors were related to capability, including (1) knowledge and acceptability of task shifting rationale; (2) dynamic role boundaries; and (3) technical skills to perform biopsies and aspirations. Five factors were related to motivation, including (4) beliefs about task shifting consequences, such as efficiency, quality and patient satisfaction; (5) beliefs about capabilities, such as technical, communicative and emotional skills; (6) job satisfaction and esteem; (7) organisational culture, such as team optimism; and (8) emotions, such as fear of informal nurse hierarchy and envy. The last two factors were related to opportunity, including (9) project planning and leadership, and voluntariness; and (10) patient preferences.

**Conclusion:**

Task shifting from doctors to nurses in specialised healthcare requires not only development of technical skills but also complex changes in organisation, clinical routines and role identity. Educational and organisational interventions to build a team-oriented culture could potentially increase the possibility of successful task shifting and stimulate nurses to take on untraditional responsibilities. Environmental restructuring to support doctors using their time in activities only doctors can perform may be needed to realise potential efficiency gains.

## Background

High-income countries face increased demand for healthcare in general as well as for more specific and more intensive care. Long waiting times and spiralling healthcare costs have resulted in calls for improved access to quality care and more effective and efficient organisation of service provision and delivery [1]. One way of addressing these challenges is to reallocate specific tasks and responsibilities between and within groups of healthcare professionals [2]. Task shifting is introduced in various high-income settings such as the Netherlands, UK, New Zealand, Australia, Canada and the U.S. as a means of dealing with an anticipated personnel shortage. A move towards workforce reconfiguration is at the same time situated in the context of public service reforms that aim to increase flexibility in working practices and service organisation, decentralise health services from specialised to less specialised care and curb healthcare expenditures [3–6]. Evidence on quality, safety, effectiveness and efficiency is, however, mixed and depends on factors such as the specific nature of role revision, type of clinicians, organisational redesign, healthcare setting and clinical area as well as the broader financial and legal context [4–7]. Further, while professional role flexibility is an important element of workforce modernisation, role boundary disputes may emerge because of role blurring and professional resistance [8]. Development of new roles for healthcare professionals can allow greater scope for multi-skilling and overlapping responsibilities, but is also associated with concerns about professional identities. Thus, attempts to implement task shifting interventions may be constrained due to boundary competition between or within groups [8–12].

Research into the factors that possibly facilitate or hinder implementation of task shifting initiatives is likely to yield insights that can inform future decisions on the feasible solutions which may apply in different settings. Until now, a great deal of research focusing upon healthcare task shifting implementation examines substitution of doctors by nurses in primary care [8, 13]. Such vertical task shifting, defined as “a process whereby specific tasks are moved, where appropriate, to health workers with shorter training and fewer qualifications”, is expected to ease bottlenecks in service delivery and make more efficient use of existing human resources [2, 6]. Nevertheless, studies have suggested that the implementation of new professional roles requires organisational redesign as well as reframing of professionalism and professional boundaries to enable task reallocation in practice [4].

Relatively less research is available on facilitators and barriers to task shifting from medical doctors to nurses in specialised hospital settings, i.e. settings that are characterised by health personnel performing highly medicalised tasks that involve access to restricted technology and are associated with physical risk to the patient [4], but see [13–15]. It has been argued, however, that while traditional professional hierarchy may be challenged by modernisation where treatment is less complex and more routine, hospital doctors are likely to continue their dominance within the complexity of diagnosis and treatment [10]. Where the professions concerned enjoy established specialist roles, the boundaries are perceived as less vulnerable. Overt boundary disputes between the professions may not be evident [8].

The need to adapt task shifting to the local setting and to understand why interventions may succeed in some settings and not in others, has led scholars to call for micro-level studies and evidence focused on process, context and mechanism in the specific setting [5, 10]. In the following, we report findings from a micro-level study of a task shifting intervention in a Norwegian haematological university hospital department. The intervention involved an “up-skilling” of nurses, introducing them to tasks currently done by medical doctors, such as performing bone marrow aspiration and biopsies. Bone marrow aspiration, the removal of bone marrow fluid, and bone marrow biopsy, which involves the removal of a core from the bone, are key diagnostic tests for patients with various haematological diseases (including leukaemia and lymphoma). Bone marrow examination is needed for disease classification and treatment, and is further used to assess disease response to various treatments [16, 17]. It is still uncommon internationally for nurses to perform these procedures [18] and there is limited research available for evaluations on task shifting in this context. Two previous studies analysed quality and patient satisfaction and concluded that with adequate planning, training and practice, nurses obtain bone marrow specimens of sufficient quality to permit diagnosis [16, 17, 19]. High level of patient satisfaction was demonstrated [20].

The aim of the present study was to identify factors that were perceived by health professionals to influence implementation of vertical task shifting from medical doctors to nurses in this specialised hospital setting. In general, implementing new practices in an organisation requires changes in both individual and collective behaviour. Behaviour change theory provides a conceptual framework to categorise potentially modifiable factors that influence behaviour change, understand mechanisms of change and inform implementation interventions [21]. We applied two tools for understanding behaviour, the Capability, Opportunity and Motivation behavioural model (COM-B) and the Theoretical Domains Framework (TDF(v2)), to gain improved understanding of task shifting implementability and pinpoint interventions that could possibly ease implementation. The theoretical approach is described in greater detail below.

## Methods

### Design

This study used a retrospective qualitative design and investigated perceptions of task shifting implementability among health professionals that were familiar with task shifting. Semi-structured individual interviews and a focus group discussion were undertaken in 2017 and theory-driven thematic analysis was deployed to systematically organise data into a structured format.

### Setting

Norwegian healthcare is universal and tax-financed, and 8,9% of GDP is spent on health [22]. While primary healthcare and social services are organised at the municipality level, an independent local administrative level, specialised healthcare is subject to national governance. Specialised healthcare is administered by four regional health enterprises and is free at the point of access. Increased demand for care, waiting list problems, economic pressure, prospected personnel shortage and continuous technological changes challenge the current organising and management of healthcare services. Recent white papers have pointed to reallocation of tasks as a strategy to deal with expected personnel shortages and increasing demand for health services [23, 24].

According to the Norwegian Health Personnel Act (1999), health personnel “shall conduct their work in accordance with the requirements to professional responsibility and diligent care that can be expected based on their qualifications, the nature of their work and the situation in general”. Health personnel may assign certain tasks to other personnel if it is considered safe to do so based on the nature of the assigned task, the qualification of the assigned personnel and the guidance that is being provided. Unlike other countries, Norway has not yet formally introduced an advanced nursing role.

The task shifting initiative analysed in this study was initiated in 2016 in a department that consisted of approximately 120 clinicians, the ratio between doctors and nurses 1:4. Task shifting was introduced to meet challenges of turnover among nurses and increase work attractiveness by creating new career opportunities and further, to increase effectiveness and efficiency in service delivery. Five nurses were trained in performing bone marrow aspirations and biopsies. The final responsibility for these tasks was not delegated but remained with the medical doctor.

### Theory

Implementation of new practices, such as task shifting, depends on actual behaviour change. We wanted a theoretical driven analysis of the perceived barriers to and facilitators of task shifting and utilised the COM-B model. This is a theory-based model that is developed in order to understand the nature of the behaviour to be changed and thus provide a basis for designing interventions aimed at behaviour change [25]. The model theorises how three conditions, namely capability, opportunity and motivation interact to produce behaviour. *Capability* is the individual’s psychological and physical capacity to engage in the activity, and include reasoning, knowledge and skills. *Opportunity* is physical and cultural-social factors such as environmental and organisational context and resources, that lie outside the individual that make behaviour possible or prompt it. *Motivation* is the reflective and automatic processes that direct behaviour. These three conditions can potentially influence each other in different ways. Capability and opportunity can influence motivation, and enacting a behaviour can alter capability, motivation and opportunity.

**Fig. 1:**
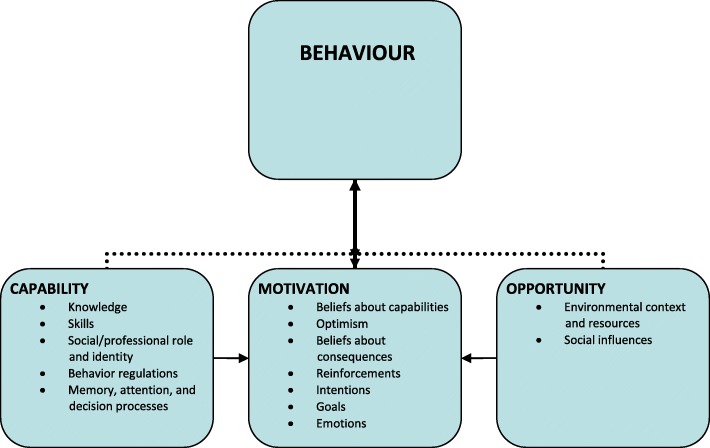
Analytical framework

To provide an even more granular understanding of these conditions, the Theoretical Domains Framework (TDF) was used [26]. TDF was originally developed as a theoretical framework to view the cognitive, affective, social and environmental influences on behaviour change. The version utilised in this study categorises 14 domains relevant to behavioural change, referred to in the following as TDF(v2) [27]. The domains include: Knowledge; skills; professional role and identity; beliefs about capabilities; optimism; beliefs about consequences; reinforcement; intentions; goals; memory; environmental context; social influences; emotions; and behavioural regulation. The domains were used to provide sub-divisions of capability, opportunity and motivation. The combined framework provided a basis for analysing the target behaviour in our specific context and mapping health professionals’ perceptions of which factors and mechanisms are working for and against task shifting. The relationship between the COM-B components, subdivided into relevant TDF(v2) domains, is illustrated in Fig. 1. Further, we wanted to determine interventions likely to be required to implement task shifting and employed a classification of intervention functions aimed at addressing deficits in capabilities, opportunities or motivation developed by Michie et al. 2011 [25]. The relationship between the COM-B components, the TDF(v2) domains and the intervention functions is illustrated in Table 1.

**Table 1:** Relationships between components, domains and intervention functions

COM-B component	TDF (v2) domain	Intervention functions
CAPABILITY - Psychological	Knowledge Professional role and identity	Education, training and enablement
- Physical	Skills	Training, enablement
OPPORTUNITY - Physical	Environmental context and resources	Restriction, environmental restructuring, enablement
- Social	Social influences	Restriction, environmental restructuring, enablement
MOTIVATION - Reflective	Beliefs about consequences, capabilities, reinforcement, optimism	Education, persuasion, incentivation, coercion
- Automatic	Emotions	Persuasion, incentivation, coercion, environmental restructuring, modelling, enablement

### Participants

We used purposive sampling to include relevant health professionals who were familiar with the term task shift and therefore were assumed to contribute with useful information, views or experiences on the topic. A total of 17 doctors (*n* = 3) and nurses (*n* = 14) were invited and included in the study. None declined the invitation to participate. The participants varied in experience, clinical and administrative roles, and education (see Table 2). All the doctors and three of the nurses were participants in the task shifting intervention project. Two of the nurses that participated in the intervention project were not included in our study because they were on leave. Informed written consent to participate in this study was obtained from all participants.

**Table 2:** Study participants’ profession and years of experience

Profession and years of experience	
Nurse < 1 year’s experience	2
Nurse > 2 years’ experience	3
Nurse > 3 years’ experience	5
Nurse > 10 years’ experience	4
Doctor (resident)	1
Doctor (senior consultant)	2
Total	17

### Data collection

Participants were asked open questions covering capabilities, opportunities and motivation. The questions allowed for flexibility in exploring different factors potentially influencing implementation of task shifting. A total of 11 medical doctors (*n* = 3) and nurses (*n* = 8) were individually interviewed. The interviews were conducted in conjunction with the interviewees` working hours and took place at the workplace. Interviews lasted from 30 to 60 min.

One focus group (*n* = 6, all nurses) discussion took place after all individual interviews were completed. None of the participants had taken part in the individual interviews. The focus group was initiated to explore if interaction between the participants provided new information about participants’ beliefs and to improve the validity of the data resulting from the individual interviews. It was initially intended to arrange two focus groups. However, focus group data repeated what was expressed in interview data and did not add to the information already gathered. We concluded that data saturation was reached and decided that further data collection was unnecessary. Individual interviews and focus group were conducted by one of the authors (AEL).

### Analysis

All interviews and focus group data were audio-recorded, transcribed verbatim and analysed using theory-driven thematic content analysis [28]. Data content was identified referring to theoretical COM-B components and TDF-domains and were organised into sub-domains. Text units with statements were entered into tables. Both authors independently coded data into domains and sub-domains and the coding was discussed among the authors to obtain satisfactory reliability. The participants were given the possibility to read a copy of data transcripts and to provide feedback.

## Results

The participants identified ten factors that they believed may influence task shifting implementation, including (1) knowledge of and acceptability of task shifting rationale; (2) dynamic role boundaries; (3) technical skills to perform biopsies and aspirations; (4) beliefs about consequences such as efficiency, quality and patient satisfaction; (5) beliefs about capabilities such as technical, communicative and emotional skills; (6) job satisfaction and esteem; (7) organisational culture such as team optimism; (8) emotions such as fear of informal nurse hierarchy and envy; (9) project planning and competent leadership. Including structured training, time to practice and voluntariness; and (10) patient preferences. In the following, we briefly describe the ten factors. Illustrative quotes from participants are given in Table 3.

**Table 3:** Example of quotes illustrating the different determinants perceived to influence task shifting implementability

Implementability determinants	Illustrative quotes
Knowledge of and acceptability of task-shifting rationale	• *It (the task shifting project) should be discussed with all stakeholders (…). It is not a good idea to introduce something that a group is critical towards from the beginning, as it often leads to adversity.* (Nurse1*)* • *Task shifting must be acceptable to both groups.* (Doctor2).
Dynamic role boundaries	• *Solving the tasks in the best possible and most efficient way is of highest importance, who performs them is less important.* (Doctor1) • *Sometimes we should stop and think, “what is actually nursing and what is our profession?”* (Nurse8). • *I think that doctors should perform diagnosis (…) Diagnosing is often about ruling out a lot of stuff too.* (Nurse7) • *Diagnosis and treatment – that might be our domain (…) It is easier to delegate practical things.* (Doctor3)
Technical skills to perform biopsies/aspiration	• *Performing bone marrow examination is a practical task (...) you do the procedure.* (Nurse11)
Beliefs about consequences • outcome efficiency • outcome quality • job satisfaction	• *It becomes more flexible who can perform things.* (Nurse4) • *Work will be more effective. There are usually more nurses than doctors at work.* (Nurse1) • *We can spend more time on things that just we can perform (…) taking care of the poorer patients.* (Doctor3) • *The more people that are involved with (…) a patient, the greater the risk of important information getting lost, which in turn can lead to complications.* (Doctor1) • *I also think that it (task shifting) may have an opposite effect - that people will quit because they will not do it. Some think it is tough to just be here and do the job they do.* (Nurse3) • *It is cool to learn something new but (...) you should have a good insight into the patient every single day. Then it is an advantage to have participated in nursing yourself.* (Nurse5)
Beliefs about capabilities • technical skills • communicative skills • emotional skills	• *Bone marrow biopsy (...) I think we are gentler. We are more concerned with how the patient feels.* (Nurse8).
Job satisfaction /Esteem	• *A motivational factor is (…) the feeling of climbing a kind of career ladder and getting new challenges (…) and lots of responsibility.* (Nurse8) • *It (the project) is (…) a declaration of trust.* (Nurse1)
Organisational culture • team positivity	• *It is a lot about the culture in a unit (...) Culture is actually a prerequisite fo00 success*. (Nurse1)
Emotions • fear of informal nurse hierarchy • envy	• *It becomes a kind of hierarchical structure (...) because they (the project nurses) feel that they are (...) better nurses.* (Nurse8) • *It must not turn into a situation where we set those tasks higher than (...) a traditional nursing job.* (Nurse11)
Project planning and leadership • Structured training • Time to practice • Voluntariness	• *It (the project) must definitely be agreed with and followed up by management and a reasonably clear goal must be set.* (Doctor2) • *You must have good training (…) Then you have to feel that you can handle it (the task) before you get the full responsibility.* (Doctor3) • *A prerequisite is that training is under guidance, that those who perform it are feeling safe, and that it is voluntary.* (Nurse1)
Patient preferences	• *I do not think it is about profession; it is about them (patients) wanting someone who has done it (the task) many times. A patient would be equally sceptical if a young doctor appears to be very unsure of the procedure, I think.* (Nurse4)

### Knowledge of and acceptability of task shifting rationale

The participants expressed the view that knowing *why* new routines are developed is vital to acceptability of task shifting initiatives. Understanding the rationale for behaviour change and being educated about the evidence base for task shifting was thought to contribute to acceptance of new roles and willingness to take on new tasks.

### Dynamic role boundaries

Both doctors and nurses emphasised the importance of thinking about delivery as a team providing healthcare rather than individuals or a single profession. They readily accepted that it was irrelevant who performed the various tasks as long as the work was organised competently. To define a limit for task shifting and decide which tasks that should be reallocated among doctors and nurses was, nevertheless, challenging for all participants. Some of the nurses experienced task shifting as a threat to the perceived nursing role because they feared a loss of general nursing skills. Most participants agreed, however, that an expansion of the nursing role had potential benefits and welcomed role flexibility. Role boundaries were drawn at diagnostic evaluation; while nurses should be familiar with diagnostic criteria, decisions that may have consequences for future diagnosis, treatment and care were believed to remain with the doctors.

### Technical skills to perform new tasks

The participants stressed that quality of service and patient safety should never be compromised by task shifting initiatives. They ranked practical training and evaluation as the strongest factor predicting implementability of task shifting. They were clear about the need to develop technical skills to perform satisfactory bone marrow aspirations and biopsies. They further argued that practical tasks were easier than tacit knowledge to delegate and take over from another profession.

### Beliefs about consequences

It was a common view among the participants that task shifting could lead to better and more efficient use of human resources given the doctor-nurse ratio of 1:4. Improved flexibility in the team was seen to be one of the major benefits of task shifting. The participants believed that task shifting would give the doctors more time to spend on the more advanced patient cases and provide better treatment. However, the participants also expressed concern about fragmentation of tasks. They pointed out how task fragmentation would introduce more people in the treatment team and were concerned about the consequences for treatment safety and quality. Further, it was seen as an unnecessary burden for the patient to relate to more personnel than necessary.

### Beliefs about capabilities

Both doctors and nurses expected that quality of bone-marrow biopsies and aspirations would be adequate if a trained nurse performed the procedures. Some nurses further believed that patient satisfaction would increase because it was anticipated that nurses would have more time than doctors to do the procedures. In addition, some of the participants highlighted that the nursing role accentuates specific skills and reflected upon capabilities they believed to be distinct to the nursing role relative to role of the medical doctor, such as greater communicative and emotional skills.

### Job satisfaction

A common theme during the interviews and focus group discussion was the belief that new challenges are essential for personal development at work. Most of the participants assumed that task shifting would favour role development for nurses. Some regarded task shifting as a statement of trust, which again was believed to lead to increased job motivation. It was also pointed out that new challenges, related to more advanced tasks, could give nurses considering resigning a reason to continue working at the ward. Nevertheless, some of the nurses explained that they were not enthusiastic about handing over traditional nursing tasks to less educated personnel because they wanted to “be close to the patient” and were ambivalent towards taking on more / other responsibilities. Consequently, there were worries that work overload and increased responsibilities would become a problem and thus hinder task shifting implementation.

### Organisational culture

The participants highlighted that a team culture of positivity and optimism was important in implementation efforts. In addition, task shifting was believed among both doctors and nurses to increase collaboration and team spirit and thus reinforce a supportive environment.

### Emotions

At the same time, the potential for creating a new informal nurse hierarchy was a recurrent theme in the interviews and focus group discussion. Several participants were afraid that there would be differences in status between the “super-nurses” that undertook special responsibilities and the other nurses, and that such hierarchical relationships would negatively affect the workplace. All did however, not share these views. It was pointed out that several nurses at the ward already had individual tasks in addition to their regular nursing tasks and that this fact had not resulted in a hierarchical structure among the nurses.

### Project planning and leadership

The participants emphasised the importance of project planning, competent management, and availability of time resources. Facilitation of structured training was regarded as a core leadership responsibility and was seen as an essential implementation enabler. Both doctors and nurses further believed that the assignment of new medical tasks to nurses needed to be based on voluntariness to be accepted as legitimate.

### Patient preferences

While it was recognized that patients may prefer doctors to perform invasive procedures and that patient preferences can hinder task shifting initiatives, the participants reported that patient perceptions of professional role boundaries did not seem to work against implementation in this context. The belief that patients do expect biopsies to be performed by competent personnel, but are indifferent to whether they are attended by a doctor or a nurse, was widespread among the participants.

## Discussion

Designing interventions directed at task reallocation is a novel approach to making better use of existing work force resources in different healthcare settings [3–5]. This study investigated how invasive technical procedures, such as performing bone marrow aspirations and biopsies, were delegated from medical doctors to nurses in a university hospital setting and identified factors perceived by health professionals to potentially affect implementation of the intervention. A theory-informed approach was used to gain better understanding of what may be facilitators and barriers to enabling task shifting. The COM-B model and the Theoretical Domains Framework allowed a structured way of considering how capability, opportunity or motivation were perceived as important to task shifting implementability. This is illustrated in Table 4.

**Table 4:** Relationships between components, domains, implementability determinants, and intervention functions

COM-B component	TDF (v2) domain	Implementability determinant	Relevant intervention - example
CAPABILITY - Psychological	Knowledge	Knowledge of and acceptability of task-shifting rationale	Education
	Professional role and identity	Dynamic role boundaries	Enablement
- Physical	Skills	Technical skills to perform biopsies/aspiration	Training Enablement
MOTIVATION - Reflective	Beliefs about consequences	Beliefs about consequences s.a. outcome efficiency; outcome quality, job satisfaction	Education
	Beliefs about capabilities	Beliefs about capabilities s.a. technical, communicative and emotional skills	Education
	Reinforcement	Job satisfaction/Esteem	Education, incentivation
	Optimism	Organisational culture Team positivity	Education, incentivation
- Automatic	Emotions	Fear of informal nurse hierarchy Envy	Environmental restructuring
OPPORTUNITY - Physical	Environmental context and resources	Project planning and leadership • Structured training • Time to practice • Voluntariness	Enablement Environmental restructuring
- Social	Social influences	Patient preferences	Education

Previous studies of vertical substitution in healthcare, i.e. task delegation across disciplinary boundaries where the levels of expertise and autonomy are not equivalent between workers, have described “the ambivalence of up-skilling” [5, 6, 29]. While increased complexity of tasks are welcomed as a means to derive greater job satisfaction, challenges are present, such as fear of malpractice, increased workload due to role expansion, change and uncertainty in the workplace and new hierarchical relationships. Concerns that a medical rather than a nursing focus gains dominance when the nursing role expands have been reported [30]. In addition, studies have suggested distrust and concern among medical doctors around responsibility and clinical quality and safety when nurses take over clinical tasks from doctors [4, 14, 31].

An “ambivalence of up-skilling” was equally found in our study. On the one hand, some of the participants expressed tensions that role revision brought to bear on the ideals embedded in the nursing role, such as worries about fragmentation of nursing care and a shift from a patient-centred, holistic approach to care to a medical focus. In addition, fear of an informal nurse hierarchy was expressed, although it was acknowledged that nurses might experience increased standing within in both their own group and among medical doctors. Some nurses described a reluctance to delegate their traditional work to others, and were concerned about increased workload.

On the other hand, the study demonstrates how the medical doctors’ and nurses’ willingness to renegotiate the boundaries between them was seen as one important determinant of task shifting implementation. Dynamic role boundaries in this setting were perceived by the participants to benefit both disciplines. Flexibility was thought to increase nurses’ job satisfaction because they may see it as taking on more prestigious work. Medical doctors were intended to delegate time-consuming work and retain the more specialised work with patients.

Performing bone marrow biopsies and aspirations clearly requires technical and specialised skills. The extent of delegation was linked to performing technical procedure but not to ownership of medical responsibility and the ultimate responsibility remained with the medical doctor. Thus, the professional boundaries were in this sense, not crossed. This may potentially result in low level of independence experienced by the nurses and leave the nurse with insufficient autonomy and professional growth [4]. However, the participants seemed to agree that there are important differences between narrow technical skill and deeper clinical judgement and that diagnostic procedures should remain the doctor’s responsibility.

In this sense, the potential conflict in the renegotiations of the nurses’ role was not materialised; medical doctors retained knowledge and power, which they did not wish to share. The nurses were assigned routine work that did not require sophisticated tools for diagnosis. Thus, the new role of the nurses did not present a threat to the work of doctors. At the same time, the nurses defended their unique ability to provide care whilst at the same time seeking to expand their role boundaries into the medical domain. Accordingly, workforce change appeared to be more consensual than conflict-oriented.

### Implications and further research

The COM-B model has been used to describe sources of behaviour that can be linked to different intervention functions that can be applied in order to change behaviour [32]. The participants in this study highlighted the significance of targeting both psychological and physical capabilities, such as knowledge, role boundaries, and technical skills, as well as reflective and automatic motivation such as beliefs about consequences and capabilities, job satisfaction, organisational culture, and negative emotions. Further, the participants emphasised that contextual factors providing opportunities, such as project planning and leadership, and patient preferences, were important to target. A range of interventions was described as illustrations of relevant interventions strategies, including: Providing knowledge of task shifting goal and rationale; training nurses in performing bone marrow aspiration and biopsy; offering time to practice.

However, it could be argued that task shifting also requires targeting psychological capabilities related to reframing of professional roles, and opportunities related to reallocation of traditional nursing tasks and opportunities for doctors to use time on more advanced cases. Further, negative emotions, such as fear and envy, must be dealt with. Increasing psychological capability and organisational opportunity could potentially be addressed through interventions that target education, persuasion and environmental restructuring. Educational and organisational interventions to build a team-oriented culture may increase the possibility of successful task shifting. Organisational restructuring may likewise be necessary to enable efficiency gains resulting from medical doctors using their time in activities only doctors can perform.

Further studies are necessary to evaluate whether task shifting in this context is feasible, effective and cost-effective, safe and does not have any unwanted side effects. For example, we did not include patient views in the present study and we do not know whether patients welcome task shifting in this specific context. Further, we have pointed out how task shifting may require increased teamwork in the workplace; however, the development of a team may take time and could restrict efficiency. Moreover, task shifting may increase recruitment of nurses but fewer nurses will be available for traditional nursing tasks and increased workload may be experienced. The consequences of task shifting regarding nursing shortage remain to be seen.

### Strengths and limitations

The combined use of the COM-B model and the Theoretical Domains Framework (TDF(v2)) provides an approach for organising evidence on key factors perceived by health professionals to influence task shifting implementation. This is a strength of this study. It enables a more rigorous and consistent discussion of evidence and provides possible comparison with other evidence on and theories of implementation. Healthcare implementation involves complex interdependencies between factors and there is a need for research on mechanisms that determine whether an intervention will be successful in a particular setting [33]. Making explicit theoretical assumptions can offer a useful framework for considering such mechanisms across settings. Use of behaviour change theory provides relevant concepts and explanations to inform the study. The specified key constructs and relationships aid systematic identification of factors that were perceived by our participants to contribute to change in the target behaviour and the subsequent design of interventions that address those factors [21, 25]. The theoretical framework used in this study takes individual behaviour as its methodological point of departure and focuses on individual perceptions, knowledge and skills, attitudes and motivation. However, it recognizes a range of factors that lie outside the individual that may act as barriers or enablers to behaviour change, such as professional norms and organisational context and culture*.*

At the same time, however, a potential limitation follows from the theoretical framework that was used. The broader organisational, economic and regulative context is not included in our study. Further, we may have overlooked factors when using preconceived categories This is a limitation that follows from the choice of using a theory-driven thematic analysis.

Another potential limitation of this study is its generalisability. Previous studies have underscored how task shifting initiatives are context sensitive and findings from our study may not be easily transferable to other settings. We acknowledge that the study examines a specialised area, and further, that the study is small. Few doctors were included; we only invited doctors that we assumed to be familiar with the specific task shifting project. However, the theoretical informed basis of this study suggest that the factors perceived to affect ask shifting implementability reported here are likely to be relevant across contexts. Many of the conclusions of this study are not specific to task shifting in a haematological department but rather have to do with task reallocation between medical doctors and nurses in highly specialised healthcare.

This study investigates factors perceived to have influence upon implementation of task shifting. The participants’ views and perceptions are subjective and we cannot know whether the participants intentionally or unintentionally framed the issues under investigation in ways that systematically influence the results of the study. Further, because the participants may be biased in their views about the problem we cannot know whether the factors identified as facilitators and barriers to implementation will be identified as such in actual practice.

## Conclusion

This study has demonstrated how factors associated with capability, opportunity and motivation were perceived by health professionals to affect implementability of doctor-to-nurse task shifting in a specialised hospital setting. Findings suggest that delegation of highly medicalised tasks that involve access to restricted technology and are associated with physical risk to the patient requires not only development of technical skills but also complex changes in organisation, clinical routines and role identity. Dynamic role boundaries were perceived to benefit both disciplines. Educational and organisational interventions to build a team-oriented culture could potentially increase the possibility of successful task shifting and stimulate nurses to take on untraditional responsibilities. Environmental restructuring may be necessary to enable efficiency gains resulting from medical doctors using their time in activities only doctors can perform. These findings offer a theory-informed basis for further developing task shifting interventions and implementation.

## Abbreviations

COM-B: The Capability, Opportunity and Motivation behavioural model
TDF: The Theoretical Domains Framework
